# An Electrochemical Approach for the Selective Detection of Cancer Metabolic Creatine Biomarker with Porous Nano-Formulated CMNO Materials Decorated Glassy Carbon Electrode

**DOI:** 10.3390/s20247060

**Published:** 2020-12-10

**Authors:** Mohammed M. Rahman, Md. M. Alam, Abdullah M. Asiri, Firoz. A. D. M. Opo

**Affiliations:** 1 Center of Excellence for Advanced Materials Research (CEAMR), King Abdulaziz University, P.O. Box 80203, Jeddah 21589, Saudi Arabia; amasiri@kau.edu.sa; 2 Department of Chemical Engineering and Polymer Science, Shahjalal University of Science and Technology, Sylhet 3100, Bangladesh; mmalamsust@gmail.com; 3 Department of Biomedical Science, College of Natural Sciences, Chosun University, Chosun 61452, Korea; fadmopo@gmail.com; 4 Phytochemistry Research Laboratory, Department of Pharmacy, University of Asia Pacific, Dhaka 1000, Bangladesh

**Keywords:** porous nano-formulated CMNO nanomaterials, creatine sensor, wet-chemical technique, glassy carbon electrode, clinical research, health care field

## Abstract

The facile wet-chemical technique was used to prepare the low-dimensional nano-formulated porous mixed metal oxide nanomaterials (CuO.Mn_2_O_3_.NiO; CMNO NMs) in an alkaline medium at low temperature. Detailed structural, morphological, crystalline, and functional characterization of CMNO NMs were performed by X-ray photoelectron spectroscopy (XPS), powder X-ray diffraction (XRD), ultraviolet-visible spectroscopy (UV-vis), Fourier-transform infrared spectroscopy (FTIR), field emission scanning electron microscopy (FESEM), and energy-dispersive X-ray spectroscopy (EDS) analyses. An efficient and selective creatine (CA) sensor probe was fabricated by using CMNO NMs decorated onto glassy carbon electrode (GCE) as CMNO NMs/GCE by using Nafion adhesive (5% suspension in ethanol). The relation of current versus the concentration of CA was plotted to draw a calibration curve of the CMNO NMs/GCE sensor probe, which was found to have a very linear value (r^2^ = 0.9995) over a large dynamic range (LDR: 0.1 nM~0.1 mM) for selective CA detection. The slope of LDR by considering the active surface area of GCE (0.0316 cm^2^) was applied to estimate the sensor sensitivity (14.6308 µAµM^−1^ cm^−2^). Moreover, the detection limit (21.63 ± 0.05 pM) of CMNO MNs modified GCE was calculated from the signal/noise (S/N) ratio at 3. As a CA sensor probe, it exhibited long-term stability, good reproducibility, and fast response time in the detection of CA by electrochemical approach. Therefore, this research technique is introduced as a promising platform to develop an efficient sensor probe for cancer metabolic biomarker by using nano-formulated mixed metal oxides for biochemical as well as biomedical research for the safety of health care fields.

## 1. Introduction

Biologically, the CA is synthesized in the kidney and liver into the human body, which is later distributed through the blood circulation system. Then it is carried to the high energy demanded tissues such as the brain and skeletal muscle [1,2] in the human body. It is also available in foods such as meat and fish [3], which are commonly used in human food chains. Besides this, CA can also be synthesized in the laboratory if needed. The most important thing is that the CA performs a vital role to produce energy and control of pH of the buffer system into the living tissues. Therefore, a continuous supply of CA is necessary either through food or biosynthesis in the human body [4]. On the other hand, the deficiency of CA may cause several neurological syndromes in the human body such as autism, extrapyramidal syndrome, hypotonia, auto-mutilating behavior, delays in speech acquisition and mental retardation [5,6,7]. On considering the physiological effects of CA, it is very important to the development of a reliable and effective quantification method of CA in biological samples. Some existing conventional methods are generally used for quantification of CA in human fluids, such as colorimetry [8], spectrophotometry [9], and fluorimetry [10]. However, these conventional methods still have some drawbacks such as heavy and expensive instruments, time-consuming, costly reagents and un-portable to in situ biomarker detection. The derivative, CA riboside, is a novel and efficient metabolite of cancer metabolism in the living body. It is known as a urinary diagnostic biomarker of lung and liver cancer risk and prognosis. Besides this, CA riboside is highly positive, which is correlated with tumors and urine derived from human lung and liver cancers [11]. Recently, the electrochemical sensor based on the electrochemical method becoming popular to detect and quantification of ionic, chemical, and bio-chemical spices [12,13,14,15,16,17,18,19,20]. Besides this, the sensitivity and selectivity of the sensor probe can enhance by using various nanomaterials as well as nanocomposites as the sensing mediator [21,22,23,24,25,26]. Therefore, the aim of this research is to develop an enzyme-less sensor by applying CMNO materials onto GCE with the help of a conducting coating binder.

Nowadays, interfacial mediators such as nanomaterials, nanocomposites, and hybrid materials are used for the reliable detection of unsafe toxic chemicals as well as biological stuff [27,28,29]. Generally, CuO is a p-type semiconductor having an optical band-gap of 1.2 eV and electrical conductivity of 7.8 × 10^−4^ W^−1^ cm^−1^ [30]. Due to the unique electrical, magnetic and optical properties of CuO, it was found to use as a sensor, catalyst, supercapacitors, infrared filters, and magnetic storage [31]. Therefore, it is also applied to the detection of 3-chlorophenol [31], bicarbonate [32], and bilirubin [33] successively as electrochemical sensors. Recently, Mn_2_O_3_ has been investigated widely as an electrochemical sensor for its physio-electrochemical properties, including direct bang-gap energy (1.2 eV) and conductivity (10^−6^~10^3^ W^−1^ cm^−1^). These properties are highly considered in the viewpoint of electrochemical sensor applications [34,35]. Moreover, NiO is another efficient p-type semiconductor with an intrinsic band-gap of 3.7~4.0 eV. It is widely applied as sensors, supercapacitors, electronic devices, photo-catalysts, and catalysts [36,37]. It is found that CuO, Mn_2_O_3_, and NiO have strong evidenced to apply as electrochemical sensors for the detection of unsafe chemicals and biochemical for the safety of living things. Therefore, this study is introduced for the development of a selective sensor probe by using the ternary mixtures of CuO, Mn_2_O_3_, and NiO.

The prospective and promising electrochemical technique for the real-time analysis of biological samples by using CMNO nanomaterials deposited GCE were fabricated and applied to the successive detection of unsafe CA. Calibration of the sensor probe is plotted from the linear portion of current versus concentration of CA. It is used to calculate the sensor analytical parameters such as sensitivity, LDR, and DL of the proposed sensor. To the best of our knowledge, CMNO nanomaterial is used for the first time as an efficient sensor substrate for the selective detection of CA in the phosphate buffer solution. Besides this, the sensitivity, reproducibility, detection limit, response time, convenience with low cost, simplicity, and capability of rapid diagnosis are analyzed with the fabricated CMNO NMs/GCE sensor probe for CA detection. Therefore, this sensor probe fabrication would be an excellent approach for the development of reliable and selective CA detection by using nano-formulated mixed metal oxides or hybrid materials in biomedical and health care fields on a broad scale.

## 2. Experimental

### 2.1. Materials and Methods

To prepare the nano-formulated CMNO NMs, the precursors NiSO_4_.6H_2_O, MnSO_4_.H_2_O, and CuCl_2_.2H_2_O were purchased from Merck, Germany. For this experiment, L(+)-lactic acid, D-glucose, folic acid, cholesterol, choline, L-glutamic acid, dopamine, L(+)-aspartic acid, ascorbic acid, CA, 5% Nafion suspension, mono- and disodium phosphate were purchased from Sigma-Aldrich (Sigma-Aldrich Corp., St. Louis, MO, US) company. An XPS (Thermo Scientific with A1 K-ɑ1 radiation sources and a beam spot size of 300.0 µm, which is performed at 200.0 eV, and 10^−8^ Torr pressure) was utilized for the CMNO NMs to evaluate the respective quantitative and chemical state information from the surface of nanomaterials. The morphology and elemental compositions of CMNO NMs were simultaneously investigated by using FESEM (JEOL, JSM-7600F, Tokyo, Japan) equipped with EDS. The functional group and photo-absorbance of CMNO nanomaterials were evaluated by using Thermo Scientific NICOLET ISSO FTIR (Thermo Scientific spectrometer). The phase crystallinity and grain size of CMNO NMs were evaluated by XRD analysis. Band-gap energy was calculated for CMNO NMs by using UV-visible spectroscopy. The proposed sensor for selective creatine was fabricated with CMNO NMs onto GCE with a conducting binding agent (5% Nafion). It was used to detect the CA in the phosphate buffer medium successively. To fabricate the sensor, Keithley electrometer (6517A, Chicago IL, USA) is used as the source of constant potential supply to analyze the current response with the injected analyte by fabricated sensor probe. It is stated that the fabricated sensor is analyzed with the two electrodes systems.

### 2.2. Synthesis of CMNO Nanomaterials

For the synthesis of CMNO NMs, NiSO_4_.6H_2_O, MnSO_4_.H_2_O, and CuCl_2_.2H_2_O were used to prepare 0.1 M solutions of each in three different volumetric flasks of 100.0 mL in deionized water, and 50.0 mL of obtained solution from each flask taken into a 250.0 mL beaker. Then, the beaker kept on a hot heater at 80 °C facilitated a magnetic stirring system. After this, the prepared 0.1 M NH_4_OH alkaline solution was added dropwise into the beaker to raise pH up-to 10.5. Due to the addition of the alkali solution, the pH increased, and metal ions precipitated as metal-hydroxides. It assumed that the metal ions precipitated quantitatively as Cu(OH)_2_.Mn(OH)_2_.Ni(OH)_2_ at 10.5 pH. The corresponding reactions are illustrated below.

NH_4_OH_(l)_⇆NH_4_^+^_(aq)_ + OH^−^_(aq)_ (1)

CuCl_2(s)_→Cu^2+^_(aq)_ + 2Cl^−^_(aq)_ (2)

MnSO_4(s)_→Mn^2+^_(aq)_ + SO_4_^2−^_(aq)_ (3)

NiSO_4(s)_→Ni^2+^_(aq)_ + SO_4_^2−^_(aq)_ (4)

Cu^2+^_(aq)_+ Mn^2+^_(aq)_+ Ni^2+^_(aq)_ + 6OH^-^_(aq)_ + nH_2_O⇆Cu(OH)_2_●Mn(OH)_2_●Ni(OH)_2(s)_· nH_2_O ↓ (5)

After the completion of co-precipitation, the beaker was kept for several hours with magnetic stirring and the same temperature on the heater. Then the obtained nanomaterials were separated by filtration from reaction alkaline medium and followed by washing with deionized water successively. Then it kept an oven at 120 °C overnight to dry. Next, the dry nanocrystals were subjected to calcine into a muffle furnace at 500 °C around 5 h in the flow of oxygen. The metal-hydroxides are oxidized and formed pure metal oxides. The reactions involved in the muffle furnace are as follows:

Cu(OH)_2_·Mn(OH)_2_·Ni(OH)_2(s)_ + O_2_→CuO·NiO·Mn_2_O_3_ + 3H_2_O_(v)_ (6)

### 2.3. Fabrication of GCE by CMNO Nanomaterials

The fabrication of GCE was a sensitive and important part of this research work for the detection of CA. At the beginning of the modification process, a slurry of CMNO NMs (0.05 mg) in ethanol had been prepared and very carefully deposited/layered on the flat surface of GCE to form a thin-film of nano-formulated CMNO NMs. Then it was kept in the laboratory at ambient temperature to dry the surfaces. As per the requirement of this study, adequate stability of the CMNO NMs deposited layer onto GCE was required. Therefore, 0.1 μL Nafion as an adhesive was added onto the CMNO fabricated layer to make the final modification of GCE. This was placed in an oven at 35 °C for an hour to dry it completely. As a result, the modified GCE was found more stable in an aqueous medium during the electrochemical investigation. The proposed sensor was measured through an electrometer (Keithley electrometer, 6517A, Chicago IL, USA) by connecting the CMNO NMs/GCE as working and Pt-wire as the counter electrodes. To characterize the electrochemical performances of the chemical sensor, the CA was diluted in deionized water to make various solutions, ranging serially from 1.0 mM to 0.1 nM. A calibration plot (plotted from the current versus concentration of CA) was used to estimate the analytical performances of the proposed CA sensor, including selectivity, LDR, and DL. During the I–V measurement, the phosphate buffer solution in the measuring electrochemical cell was kept constant as 10.0 mL throughout each experiment.

## 3. Results and Discussions

### 3.1. Characterization of CMNO Nanomaterials

**Figure 1:**
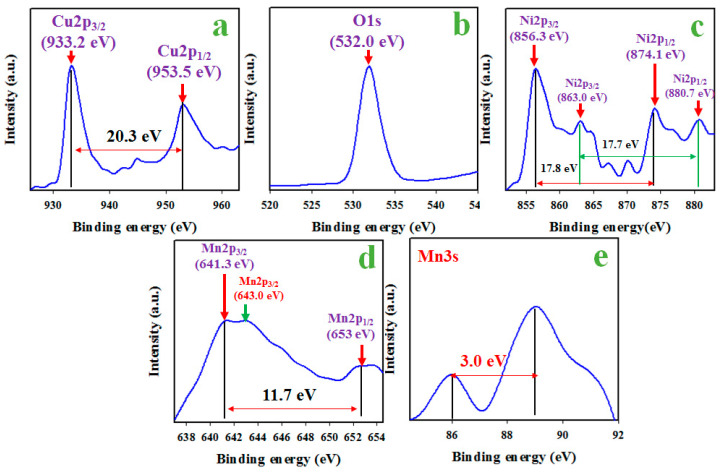
XPS analysis of low-dimensional nano-formulated porous mixed metal oxide nanomaterials (CMNO NMs) by X-ray photoelectron spectroscopy. The XPS spectrum of (**a**) spin-orbital of Cu2p level, (**b**) O1s orbit, (**c**) high-resolution XPS spectrum of Ni2p level, and (**d**) Mn2p spin orbits and (**e**) Mn3s level.

To investigate the empirical formula and binding energies of atoms that existing in the nano-formulated CMNO NMs, the XPS analysis was executed and presented in Figure 1. The resulting XPS spectrum showed the peaks corresponding with Cu, Ni, O and Mn elements. The illustrated Cu2p spin–orbit shows two peaks at 933.2 and 953.5 eV with respect to Cu2p_3/2_ and Cu2p_1/2_ in CuO having a separation of 20.3 eV, which is shown in Figure 1a. This value of spin energy separation confirms the existence of Cu^2+^ oxidation in prepared nanomaterials [38,39]. A peak of O1s at 532.0 eV illustrates in Figure 1b and can be defined as the lattice oxygen with the ionization O^2-^ in sample CMNO NMs [40,41].

Figure 1c represents the high-resolution of the Ni2p spectrum. The obtained peaks are found at 856.3 and 874.1 eV for Ni2p_3/2_ and Ni2p_1/2_ spin orbitals, respectively, which has an energy difference of 17.8 eV. Similarly, two satellite peaks of Ni2p level are assigned as 863.0 eV for Ni2p3_/2_ and 880.7 eV for Ni2p_1/2,_ respectively, and the binding energy deterrence between these two satellite peaks of Ni2P is 17.7 eV. Therefore, the satellites and original peaks positions of Ni2p confirm the existence of Ni^2+^ ionization [42,43]. As demonstrated in Figure 1d, the Mn2p XPS spectrum shows the two peaks located at 641.3 eV for Mn2p_3/2_ and 653.0 eV for Mn2p_1/2,_ respectively. The binding energy difference of Ni2p level is 11.7 eV, which is a characteristic value representing the oxidation state of Mn^3+^. An associated satellite peak for Mn2p illustrates in Figure 1d at 643.0 eV, which is responsible for the oxidation state of Mn^4+^. Therefore, the average oxidation state of Mn confirms from Mn3s orbit, which is demonstrated in Figure 1e. The spin energy separation in Mn2s orbit is shown at 3.0 eV. Therefore, it can predict that the average ionization of Mn is +3 [44,45,46,47].

**Figure 2:**
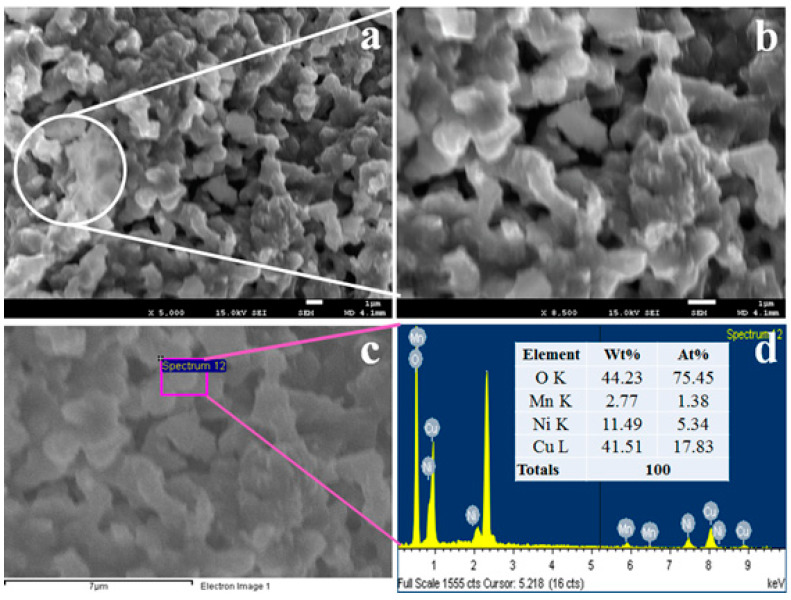
The FESEM and EDS analyses of CMNO nanomaterials. (**a**,**b**) The structural morphology of synthesized CMNO nanomaterials and (**c**,**d**) the elemental analysis of prepared CMNO NMs.

To investigate the structural shape of prepared CMNO NMs, FESEM analysis was performed as the low to high-resolution images of FESEM represent in Figure 2a,b. As shown in Figure 2a,b, the molecule of metal oxides aggregate in an irregular way to form nanomaterial in shape. According to the EDS analysis report in Figure 2c, a similar result was repeated. The elemental compositions obtained from the EDS report, as illustrated in Figure 2d, are Mn (2.77%), O (44.23%), Ni (11.49%), and Cu (41.51%) as a weight percentage.

**Figure 3:**
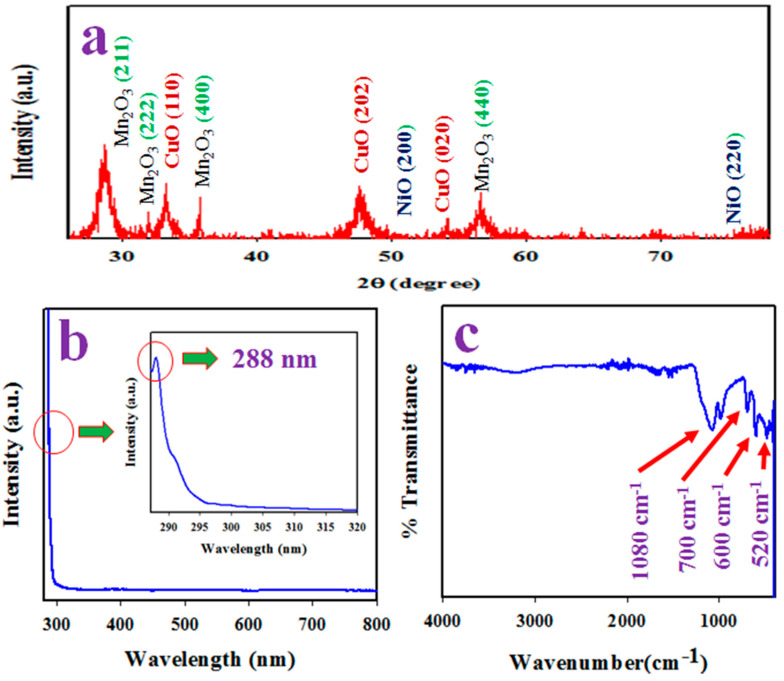
XRD, UV-vis and FTIR analysis of CMNO nanomaterials. (**a**) Crystallinity of CMNO NMs by XRD analysis, (**b**) UV-vis absorbance analysis and (**c**) the existing functional group investigation using FTIR analysis.

XRD analysis was performed to identify the phase crystallinity of synthesized CMNO NMs. The XRD pattern shows the several peaks of CuO as (110), (202), and (020) are illustrated in Figure 3a, which matched with the JCPDS no 048-1548 [48,49]. The crystalline phases of Mn_2_O_3_ such (211), (222), (400), and (440) are identified in the XRD spectrum, which is illustrated in Figure 3a. The resultant planes of Mn_2_O_3_ are found similar to JCPDS no 041-1442 [50,51]. The NiO shows less intensive peaks of (200) and (220) as presented in Figure 3a, which is matched in JCPDS no. 047-1049 [52,53]. The average grain size of CMNO NMs is estimated by applying Debye-Scherer’s formula according to Equation (7). It is found to be 10.71 nm at peak Mn_2_O_3_ (211).

D = 0.9λ/(βcosθ)(7)

Here, λ is the wavelength of X-ray radiation (1.5418 A°), β is full width at half (FWHM) of the peak at diffracted angle θ.

To measure the band-gap energy, the synthesized CMNO nanomaterial was analyzed by UV-visible spectroscopy in the range of 265 nm to 800 nm, which is presented in Figure 3b. As shown in Figure 3b, an absorption band was found at 288.0 nm, which corresponds to the band-gap energy of 4.31 eV [54,55]. This was calculated from Tauc’s Equation (E_bg_ = 1240/λ_max_). The functional groups existing of prepared CMNO NMs were identified by FTIR analysis in the range of 400 to 4000 cm^−1^, which was demonstrated in Figure 3c. Peaks at 520, 600, 700 and 1080 cm^−1^ were observed. The peak positioned at 520, 600, 700 cm^−1^ was described for the metal–oxygen (Cu-O, Ni-O and Mn-O) bond stretching vibration, and 1080 cm^−1^ was associated with OH- bending vibration [56,57]. The observed peaks at 600 and 700 cm^−1^ were responsible for bond stretching of M-O bond formation [58].

### 3.2. Application of CA Sensor with CMNO NMs/GCE

An electrochemical sensor for selective CA fabricated by using wet-chemically prepared CMNO nanomaterials was deposited onto GCE as a thin layer with 5% Nafion adhesive. The purpose of using Nafion as a binder was not only to enhance the binding strength it also increased the conductivity and electron transfer rate of the prepared sensor [59,60]. Then, the assembled CA sensor based on CMNO NMs/GCE was subjected to detect CA in a phosphate buffer medium of pH 7.0. As per the literature search, CMNO NMs were applied for the first time to develop a CA sensor probe. No reports regarding this CA sensor probe development were found in any scientific report. In the electrochemical analysis of CA, the holding time on the Keithley electrometer was set as 1 s as constant throughout this study for the investigation of analytical parameters.

**Figure 4:**
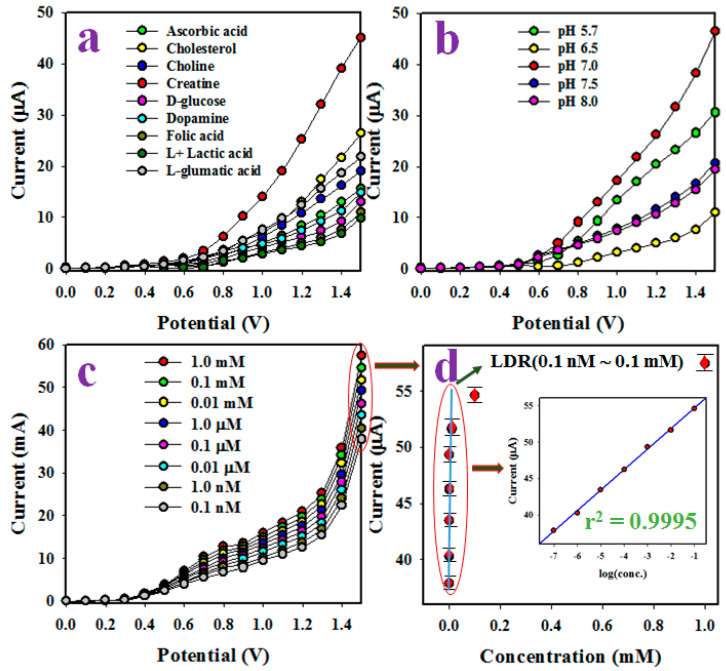
Characterization and evaluation of analytical performances of CMNO sensor probe for the detection of creatine (CA) chemical. (**a**) Selectivity analysis, (**b**) pH optimization, (**c**) electrochemical responses based on concentration variation of CA solution, and (**d**) calibration curve. Inset ((log (conc.) vs. current).

For the characterization of the fabricated sensor probe, several biochemicals were analyzed at 0.1 μM concentration (similar concentration) in applied potential (Ranges: 0~+1.5 V) in a phosphate buffer medium (pH 7.0). Figure 4a represents the electrochemical responses of D-glucose, folic acid, L(+)-lactic acid, cholesterol, choline, L(+)-aspartic acid, L-glutamic acid, dopamine, ascorbic acid, and finally CA. The CA shows the highest electrochemical response compared to other chemicals. Considering this highest electrochemical response of CA, it is found as a selective chemical with the CMNO sensor probe by using the electrochemical method. It is not logical that the CMNO NMs layer onto GCE will show the equal electrochemical activities in the phosphate buffer solutions of all pH values in the presence of CA. Therefore, the pH optimization of the buffer is necessary to obtain the maximum I–V response in this electrochemical analysis. The pH optimization performances were investigated in the range of pH (5.7–8.0) of the buffer solutions, which is presented in Figure 4b. pH 7.0 is found as optimum for CMNO NMs on GCE in the electrochemical analysis of CA, which is compared to another pH.

Then, the CA sensor with CMNO NMs/GCE was applied to the analysis of CA in a range of 1.0 mM to 0.1 nM (Potential: 0~+1.5 V) presented in Figure 4c. As obtained in Figure 4c, the electrochemically analyzed data of CA is increased with the enhancement of CA concentration. The I–V outcomes are found distinct, which is gradually enhanced from low to a high concentration of CA. The comparative result has been cited previously in the electrochemical detection of various chemicals and bio-chemicals [61,62]. The calibration of the CA sensor is executed in Figure 4d by applying the current data from Figure 4c (Potential +1.5 V). This current versus CA concentration plot is found linear over the concentration of 0.1nM to 0.1 mM, which is denoted as the linear dynamic range (LDR) of CA detection in the phosphate buffer solution. As illustrated in Figure 4d, the current density data are regularly distributed on the line of the calibration plot. These outcomes provide good evidence about the reliability of the method. Thus, the obtained LDR is found really wider ranges for CA detection. The slope of LDR and active surface area of GCE (0.0316 cm^2^) were used to calculate the CA sensor sensitivity as 14.6308 µA µM^−1^ cm^−2^. It is found very good sensitivity value with this fabricated sensor probe with nano-formulated materials. The detection limit of the CA sensor is obtained using a signal/noise (S/N) ratio of 3, which is equal to 21.63 ± 0.2 pM. It is also found very good results with CMNO NMs/GCE sensor. The linearity (r^2^) of the calibration curve is calculated and found as 0.9995. The root-mean-square error (RMSE in LDR; 0.1 nM to 0.1 mM) is calculated and found as 0.001897 (uA). On the other way, the RMSE value in the full concentration ranges (0.1 nM to 1.0 mM) is also calculated and found as 11.74138 uA.

**Scheme 1:**
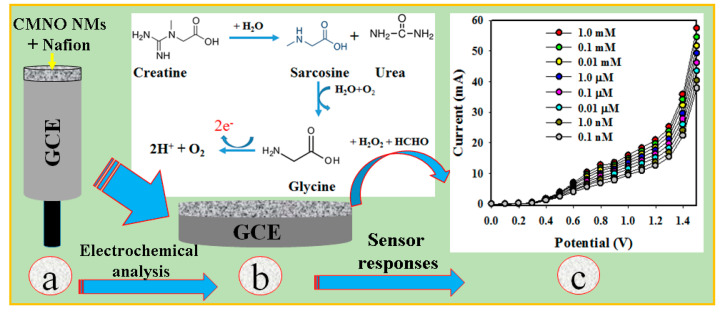
Schematic representation of CA sensing CMNO NMs/GCE. (**a**) Modification of glassy carbon electrode (GCE) with CMNO NMs, (**b**) electrochemical oxidation of CA on CMNO NMs/GCE, and (**c**) responses recorded in electrometer.

The proposed electrochemical oxidation of CA represents in Scheme 1, and Scheme 1a presents the modification process of GCE with CMNO NMs, Scheme 1b shows the oxidation of CA, and Scheme 1c illustrates the data recording system in Keithley electrometer. The nano-formulated oxidizes on CMNO NMs/GCE at the applied potential to sarcosine and urea as illustrated in Scheme 1a. Further oxidation, glycine, formaldehyde and hydrogen peroxide form and subsequently, H_2_O_2_ dissociates to generate protons, electrons and oxygen. Similar electrochemical oxidation has reported earlier [63]. As observed in Scheme 1b, the electrochemical oxidation of CA produces two electrons, which are responsible for the high conductivity of the CA detection buffer medium.

**Figure 5:**
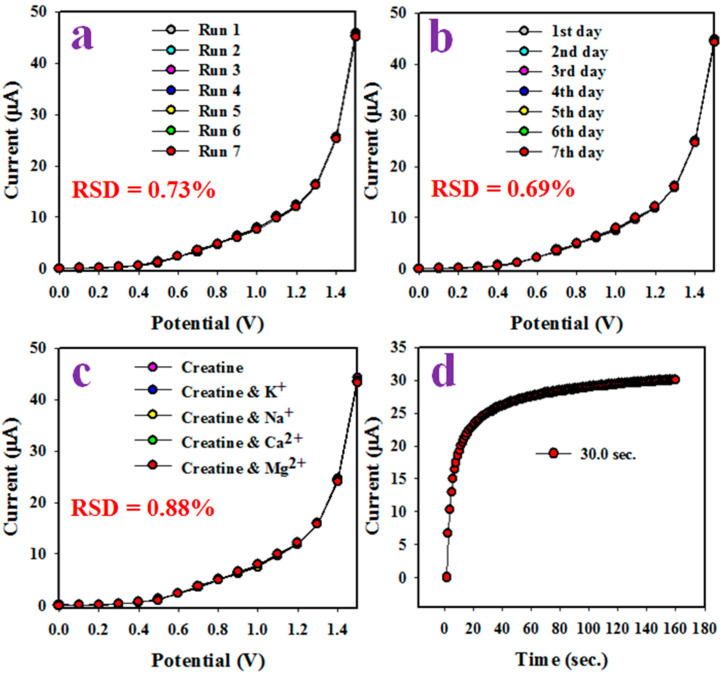
Optimization of the creatinine sensor. (**a**) Reducibility of the CA sensor, (**b**) validity of CA sensor around 7 days, (**c**) interference effect estimation in the presence of electrolytes and (**d**) response time.

The reproducibility parameter of a sensor is very important for the reliability of a sensor. Here CA sensor with CMNO NMs/GCE electrode is subjected to measure the reproducibility as well as repeatability. Therefore, this performance is executed in identical conditions in 0.1 µM CA (Potential ranges: 0~+1.5 V), which is represented in Figure 5a. As shown in Figure 5a, the seven electrochemical analyses exhibited CA detection in successive 7 h in a day are indistinguishable and almost similar responses are found. These electrochemical analyses of CA with fabricated electrodes were not significantly changed by the PBS washing of each working electrode after individual analysis. The precision of this reproducibility parameter was measured by using the terminology of relative standard deviation (RSD), which found 0.73% at potential +1.5 V. It provides the information of high precision of reproducibility. Thus, this test informs that the CA sensor probe can perform reliably in the field of its real-time application. A similar test has been performed for an extended period of seven days, which is illustrated in Figure 5b. The analogous information is obtained from Figure 5a. Thus, the evidence of this test has confirmed the long-life of the working electrode of the CA sensor (CMNO NMs/GCE) in the PBS solution. Human serum is generally contained some common ions like K^+^, Na^+^, Ca^2+^, Mg^2+^. The influences of these ions are considered in the electrochemical analyses of CA. Therefore, the CA was analyzed electrochemically in the presence of these common ions, as illustrated in Figure 5c. It can be concluded from the outcome of Figure 4c that the CA sensor has not any interfering effect of these ions in the detection of CA with CMNO NMs/GCE sensor. The response time provides information on the efficiency of a sensor probe. This test is done at 0.1 µM CA in the buffer solution of pH 7.0, which is demonstrated in Figure 5d. As it has seemed from Figure 5d, an efficient result of response time around 30.0 s is obtained.

**Figure 6:**
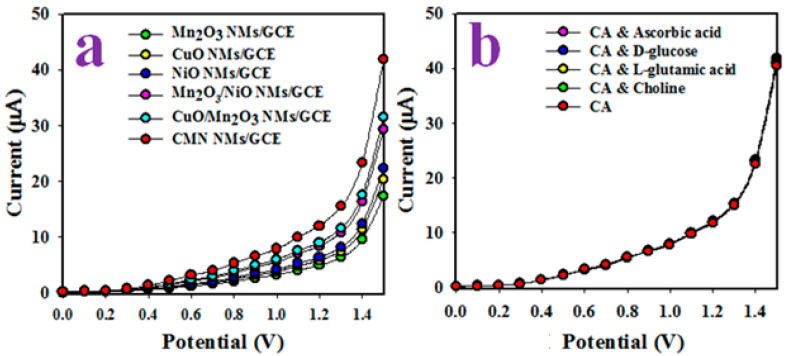
Optimization of the sensor probe. (**a**) Control experiments to evaluate the electrochemical activities of various NMs, and (**b**) interferences effect of sensor probe in the detection of CA with the presence of biochemical.

The proposed CA sensor probe was fabricated, and sensor performance was compared with ternary mixed metal oxides as well as single and binary counterparts (CuO, Mn_2_O_3_, NiO, Mn_2_O_3_/NiO, CuO/Mn_2_O_3_). Control experiments were performed to explain the uses of such various combinations. As illustrated in Figure 6a, the GCE was modified with single metal oxides (CuO, Mn_2_O_3_, and NiO) and subjected to electrochemical analysis with 0.1 µM CA detection in PBS (pH 7.0). As perceived in Figure 6a, the single metal oxides are exhibited the lower electrochemical responses, which compared to CMNO in the detection of CA. On the other hand, the binary mixture of metal oxides such as CuO/Mn_2_O_3_ NMs and Mn_2_O_3_/NiO NMs show a higher electrochemical response compared to their single metallic oxide. Finally, the ternary mixture of metal oxides (CMNO NMs) shows the highest electrochemical response compared to the single and binary-modified GCE due to the large and active surface area of CMNO.

It is exhibited due to the combinational effects of ternary metal oxides. As a result, these ternary metal oxides decorated GCE showed a better sensitivity compare to single metal oxides, as presented in Table 1 [64,65]. It has been reported that the doped metal oxides have shown better electrochemical properties than the undoped metal oxide [66,67]. As reported previously, the reactive surface area of synthesized nanomaterials is increased significantly due to the doping of nanostructured materials [68,69,70]. This is the main fact to enhance the electrochemical activity of the fabricated CA sensor probe in the detection of target CA. Another control experiment was performed to check the interference effect in the electrochemical analysis of CA in co-existence of other biochemical, as illustrated in Figure 6b. It is found that the co-existence of other biochemicals such as ascorbic acid, D-glucose, L-glutamic acid and choline with CA does not have any interference effect. Therefore, the CA sensor probe with CMNO NMs/GCE is selective to CA in electrochemical detection.

Shortly, it is concluded that the proposed CA sensor exhibited good and significant results in terms of sensitivity, LDR, and DL. Besides this, it was found as long-term stable with constant outcome in an identical condition in short response time. A comparison between similar research is represented in Table 1, in terms of sensitivity, linear dynamic range and detection limit [64,65]. It observes that the fabricated sensor probe is selective towards target CA in enzyme-less sensing with low-dimensional nano-formulated ternary CMNO NMs by using electrochemical methods, which related other reports in enzyme-less CA detection with different sensing matrixes such as nanomaterials or nanocomposites [71,72,73,74,75,76,77,78,79].

### 3.3. Real Samples Analysis

The CA sensor based on CMNO NMs/GCE was implemented for the analysis of various real biological samples such as mouse serum, human serum and rabbit serum. They were collected from the local medical store and used in accordance with ethical guidelines. Initially, the serum samples were separated and diluted (10 times) in the PBS buffer. The results are presented in Table 2. As it appears in Table 2, the analysis data were acceptable and in good agreement with the results.

## 4. Conclusions

Here, a selective chemical sensor was developed with enzyme-less CMNO NMs/GCE, which was used to detect selective CA in the phosphate buffer phase at pH 7.0. The good analytical parameters of cancer metabolic CA bio-marker sensor such as sensitivity, stability, reproducibility, linearity, linear dynamic range, and detection limit were measured, and the corresponding sensing results were found. Besides those, the target CMNO NMs/GCE sensor probe was validated and finally analyzed with biological samples such as a human serum, rabbit serum, mouse serum, human urine. It was obtained good results in this enzyme-free detection. Thus, the enzyme-less CMNO NMs/GCE sensor probe was exhibited good reproducibility in short response time, ultra-sensitivity, excellent selectivity, which provides a promising methodology for clinical cancer metabolic CA detection in biomedical and healthcare fields on a broad scales.

## Figures and Tables

**Table 1:** Comparison of CMNO-fabricated sensor probe performances for the detection of CA based on different electrode fabrication by electrochemical approaches.

Modified GCE	* DL	^#^ LDR	Sensitivity	Ref.
CdO NP/GCE	50.0 pM	0.1 nM~0.1 M	1.90 μA μM^−1^ cm^−2^	[64]
ATO-NPs/Nafion/GCE	42.0 pM	0.1 nM~1.0 mM	0.276 μA μM^−1^ cm^−2^	[65]
CMNO NMs/GCE	21.63 pM	0.1 nM~0.1 mM	14.631 μA μM^−1^ cm^−2^	This work

* DL (detection limit) and ^#^ LDR (linear dynamic range).

**Table 2:** Analyses of real biological samples with CMNO NMs/GCE sensor probe by electrochemical method.

Real Samples	Added CA conc. (µM)	Measured CA conc. ^a^ by CMNO NMs/GCE (µM)			Average Recovery ^b^ (%)	RSD ^c^ (%) (n = 3)
		R1	R2	R3		
Mouse serum	0.01	0.00992	0.00996	0.00996	99.52	0.23
Rabbit serum	0.01	0.00997	0.00994	0.00995	99.56	0.15
Human urine	0.01	0.01179	0.01182	0.01165	117.54	0.77

^a^ Mean of three repeated determination (signal to noise ratio 3) with CMNO NMs/GCE. ^b^ concentration of CA determined/concentration taken (unit: nM). ^c^ relative standard deviation value indicates precision among three repeated measurements (R1, R2, R3).
